# Adverse Pregnancy Outcomes among Adolescents in Northwest Russia: A Population Registry-Based Study

**DOI:** 10.3390/ijerph15020261

**Published:** 2018-02-03

**Authors:** Anna A. Usynina, Vitaly Postoev, Jon Øyvind Odland, Andrej M. Grjibovski

**Affiliations:** 1 Department of Community Medicine, Faculty of Health Sciences, UiT The Arctic University of Norway, Tromsø 9037, Norway; jon.oyvind.odland@uit.no; 2 Department of Neonatology and Perinatology, Northern State Medical University, 51 Troitsky Ave., Arkhangelsk 163000, Russia; 3 Department of Public Health, Health Care and Social Work, Northern State Medical University, 51 Troitsky Ave., Arkhangelsk 163000, Russia; vipostoev@yandex.ru; 4 Central Scientific Research Laboratory, Northern State Medical University, 51 Troitsky Ave., Arkhangelsk 163000, Russia; andrej.grjibovski@gmail.com; 5 Department of Public Health and Healthcare, Hygiene and Bioethics, North-Eastern Federal University, 58 Belinsky Str., Yakutsk 677000, Russia

**Keywords:** Apgar score, birth registry, low birthweight, preterm birth, Russia, stillbirth, adolescent pregnancy, very low birthweight

## Abstract

This study aimed to assess whether adolescents have an increased risk of adverse pregnancy outcomes (APO) compared to adult women. We used data on 43,327 births from the population-based Arkhangelsk County Birth Registry, Northwest Russia, for 2012–2014. The perinatal outcomes included stillbirth, preterm birth (<37 and <32 weeks), low and very low birthweight, 5 min Apgar score <7 and <4, perinatal infections, and the need for neonatal transfer to a higher-level hospital. Multivariable logistic regression was applied to assess the associations between age and APO. Altogether, 4.7% of deliveries occurred in adolescents. Both folic acid intake and multivitamin intake during pregnancy were more prevalent in adults. Adolescents were more likely to be underweight, to smoke, and to have infections of the kidney and the genital tract compared to adult women. Compared to adults, adolescents were at lower risk of low birthweight, a 5 min Apgar score <7, and need for neonatal transfer. Adolescents had no increased risk of other APO studied in the adjusted analysis, suggesting that a constellation of other factors, but not young age per se, is associated with APO in the study setting.

## 1. Introduction

Pregnancy in adolescents continues to be an important public health challenge worldwide. Approximately 11% of all births occur among 15–19-year-olds [1]. In 2014, the global birth rate in 15–19 years old adolescents was 49 per 1000 women [2]. In 2015, women aged 15–19 years gave birth to 229,715 infants in the United States [3]. The adolescent pregnancy rate in the US remains the highest among the western high-income countries and comprises 57 pregnancies per 1000 women aged 15–19 years [4]. In Europe, the pregnancy rates in adolescents aged 18 and less years vary from less than 2.0 per 1000 women aged 15–17 years in Denmark, Netherland, Finland, and Sweden to 35.5 and 29.2 per 1000 in Bulgaria and Romania, respectively [5]. In low-income countries, the adolescent pregnancy rate is several times higher; in Niger, for example, every fifth pregnancy occurs in 15–19-years-old women [6]. 

Different factors contribute to adverse pregnancy outcomes (APO). Maternal cigarette smoking [7,8], alcohol drinking [9], and drug use [8] during pregnancy increase the risk of stillbirth. Compared to healthy women, pregnant women with infections of the urinary [10,11] and genital tract [12] are at higher risk of preterm birth. Poor antenatal care [13] and maternal infections [14] are associated with early-onset infections in newborns. An antepartum urinary tract infection is an established risk factor of low birthweight (LBW), small for gestational age (GA), and perinatal death among babies born to women aged 20–29 years [11].

Earlier studies have shown that, compared to adult women, pregnant adolescents have a higher prevalence of smoking [15,16,17] and age-inappropriate education [18]. Young women are more likely to be underweight [15,19,20] and have inadequate antenatal care [15,17,18,20,21,22]. Mothers aged 16–19 years have a higher prevalence of recreational drug use and smoking compared with women aged 20–24 years [19]. The adolescents are more likely to initiate antenatal care later [22] and to have urinary tract infections [16,23]. Women aged <25 years are less likely to use dietary supplements compared to older women [24]. 

The results of studies on the associations between maternal age and pregnancy outcomes in young women are controversial. Numerous studies have reported that adolescent pregnancy is associated with preterm birth [17,18,19,20,21,22,25,26,27], neonatal mortality [17], combined perinatal and neonatal mortality [27], stillbirth [21], low Apgar score [17,22], LBW or very LBW (VLBW) [17,18,20,22,25], small for GA [18,20] or intrauterine growth restriction [21], as well as neonatal intensive care unit (NICU) admission [22]. Other studies have not found an increased risk for perinatal [15,22] and neonatal [17,20] mortality, as well as small for GA [26,27]. This heterogeneity in the results can be at least partly explained by the use of different definitions of adolescent pregnancy, the study of different age groups, and the use of different variables for adjustment that makes the comparison and interpretation of the results complicated [28], warranting further research. 

In Russia, the adolescent pregnancy rates have been decreasing over the last decades. The annual number of births per 1000 women aged 15–19 comprised 55.0, 44.8, 27.4, and 21.5 in 1990, 1995, 2000, and 2016, respectively [29]. Abortions before 12 weeks are free in Russia. In 2014, four percent of the total number of legal abortions were among adolescents aged 15–19 years [30]. Data on the prevalence of illegal abortions in adolescents is not available. 

Available data on adolescent pregnancy in Northwest Russia are scarce. Arkhangelsk County is partially located in the Arctic zone of Russia. In 2016, Arkhangelsk County had a total area of 413,100 km^2^ and a population exceeding 1.1 million [30]. People aged 15–29 years comprised 284,000 (23.4%) of the population [31]. In 2004, Grjibovski et al. [32] demonstrated that infants born to 15–19-year-olds were lighter at birth and more likely to have LBW compared to babies born to older women. From 2011 to 2013, the prevalence of pelvic inflammatory diseases in 15–17-years-old girls increased by 40%, corresponding to 84.8 per 1000 girls of that age. In 2013, salpingitis and oophoritis were revealed in 1.2% of female adolescents aged 15–17 years. As many as 1.7% of 10–14-years-old girls experienced menstrual cycle disorders [33]. In the Russian Arctic zone, 12.6% of current and former smokers began to smoke before 15 years of age [34]. Women younger than 20 years comprised 12.8% of all women who smoked before or during pregnancy [35]. 

At present, no study has assessed the perinatal outcomes in adolescent pregnancies in Northwest Russia using large population-based samples. The aim of this study was to assess whether adolescent pregnancy is associated with selected APO in Northwest Russia. 

## 2. Materials and Methods

### 2.1. Study Population and Design

This study is a retrospective registry-based study with data from the Arkhangelsk County Birth Registry (ACBR), Northwest Russia. A detailed description of the ACBR was published earlier [36]. The ACBR includes data on virtually all live- and stillbirths from 22 weeks of gestation in Arkhangelsk County from 1 January 2012. The coverage of the ACBR is 99.6%. The identity of information in medical documents, registration forms, as well as in electronic databases was approved by quality controls [36]. The ACBR contains maternal sociodemographic, lifestyle, and behavior information, data about the mother’s health before and during the pregnancy in consideration, information about the delivery and the newborn’s health. To the date, the ACBR is a systematic data collection in medical documents. Standard paper registration forms are used. The data are collected at delivery units after the birth of the child before his discharge and transfer from the hospital, or before the woman’s discharge in stillbirth or the baby’s death. Midwives and nurses or other health workers are responsible for data registration. They use both data on antenatal care and data on pregnancy outcomes documented in medical records. No additional interviews with postpartum women are used. All records from paper-based registration forms are then transferred to a depersonified electronic database [36].

For the purpose of this study, we used quality assured data on all live- and stillbirths recorded in the ACBR between 1 January 2012 and 31 December 2014 (*n* = 43,327). The number of births included in the prevalence analyses of each of the studied maternal characteristics varied, as we excluded births with missing information. To perform the prevalence analyses of APO, we excluded multiple births (*n* = 494), births prior to 22 and after 45 completed weeks of gestation (*n* = 1), and births with unknown GA at delivery (*n* = 200). Births with missing information on each of the studied maternal medical and behavior characteristics, as well as potential confounders were excluded from the study population when we performed logistic regression analyses. The number of excluded births varied, as we used several logistic models for each APO when used as the dependent variable.

GA was assessed by obstetricians and recorded in medical documents as well as in the birth registry. The assessment of GA was primarily based on first ultrasound data. In women with missed data of ultrasound examination, the data of the last menstrual period were used. Preterm births were defined as those that occurred before 37 completed weeks [37]. We used the World Health Organization’s definition of adolescent pregnancy, that is pregnancy in a woman aged 10–19 years [38]. In this study, we defined the age of the woman as her age at the time of the baby’s delivery. Women aged ≥20 years were considered as adults.

### 2.2. Outcome Variables

We used each of the studied pregnancy outcome (stillbirth, preterm birth <37 weeks, preterm birth <32 weeks, LBW, VLBW, neonatal transfer to higher-level hospital, infections specific to the perinatal period, as well as the 5 min Apgar score less than 7 and 4) as a dichotomous dependent variable. LBW and VLBW were defined as birthweight <2500 g and <1500 g, respectively [37]. We treated the variable “infections specific to the perinatal period” as a dichotomous variable that corresponded to the information on this particular issue recorded by the check-box method in the ACBR registration forms.

### 2.3. Independent Variables

In this study, the timing of the first antenatal visit, maternal smoking in pregnancy, body mass index (BMI), multivitamin and folic acid intake in pregnancy, evidence of alcohol and drug abuse, as well as infections of the kidney and the genital tract in pregnancy were used as independent variables. The first antenatal visit at 12 and more weeks of gestation was defined as late antenatal visit. Maternal education was classified as none or primary [class 1–9], secondary [class 10–11], vocational school, and higher. We calculated the BMI by dividing the weight (kg) by the height squared (m^2^). The maternal BMI at the first antenatal visit were categorized into three groups: underweight (BMI < 18.5 kg/m^2^), normal weight (BMI = 18.5–24.9 kg/m^2^) (reference group), and overweight and obese (BMI ≥ 25.0 kg/m^2^) [39]. The mothers were categorized as smokers and nonsmokers according to their smoking status during pregnancy. Evidence of maternal alcohol abuse in pregnancy was recorded as “no” or “yes”. The same was done for the variable “evidence of maternal drug abuse in pregnancy”. Nonsmokers and those who had no evidence of either alcohol or drug abuse served as reference groups. “Infections of kidney in pregnancy” and “Infections of the genital tract in pregnancy” were recorded as “no” or “yes”. We used information on International Statistical Classification of Diseases and Related Health Problems 10th revision (ICD-10) codes O23.0 and O23.5 in the ACBR for “Infections of kidney in pregnancy” and “Infections of the genital tract in pregnancy”, respectively. In registration forms, information on ICD-10 codes O23.0 and O23.5 was recorded by the check-box method. We categorized parity as primipara (reference group) and para.

### 2.4. Data Analysis

Chi-squared tests were employed to compare the distribution of the selected medical and behavior characteristics between adolescents and adult women. Multivariable logistic regression was applied to assess independent associations between adolescent age and the outcomes adjusted for potential confounders. As all studied APO are rare, odds ratios (ORs) were used as proxy estimates for relative risks. In multivariable analyses, we adjusted for maternal education, smoking, BMI, year of delivery, multivitamin and folic acid intake, infections of the kidney and the genital tract in pregnancy, and timing of the first antenatal visit. Associations between maternal age and LBW and between maternal age and VLBW were also adjusted for preterm birth. The statistical analyses were performed using IBM SPSS Statistics for Macintosh, Version 24.0. (IBM Corp, Armonk, NY, USA). 

### 2.5. Ethics Approval

The Ethical Committee of the Northern State Medical University (Arkhangelsk, Russia) approved this study (Protocol 01/02-17). The ethical approval was also obtained from the Regional Committee for Medical and Health Research Ethics in Northern Norway (2013/2300/REK Nord).

## 3. Results

Out of a total of 43,327 births, 2033 (4.7%) were from adolescents.

### 3.1. Prevalence of Medical and Behavior Characteristics in Adolescents and Adult Women

Compared with adults, adolescents were more likely to be underweight, primipara, to smoke, to have infections of the kidney and the genital tract, and less likely to take folic acid and multivitamins during pregnancy (Table 1). Young women were 2.4 times as likely as adults to initiate antenatal care after 12 weeks of gestation. There were no significant differences between the groups in the prevalence of alcohol or drug abuse.

### 3.2. Prevalence of Pregnancy Outcomes in Adolescent and Adult Women

Compared to infants born to adult women, the infants of adolescents were more likely to have LBW and required more frequent transfer to a higher-level hospital (Table 2). We did not find differences in the proportions of other studied APO between the groups of adolescents and adults.

### 3.3. Association between Maternal Age and APO

Only the variables that were significantly associated with outcomes in the bivariate analyses (Table 2) were included in the multivariable models (Table 3). In the bivariate analysis, the risk of neonatal transfer to a higher-level hospital was 16% higher in adolescents compared to adult women (Table 3). The young maternal age was associated with an increased risk of LBW. After adjusting for confounding factors, we found associations between young maternal age and decreased risk of neonatal transfer, LBW, and a 5 min Apgar score <7. 

## 4. Discussion

Adolescents had no increased risk of any studied APO. After adjustment for potential confounders, adolescents’ infants were less likely to have LBW, a 5 min Apgar score <7, and to need neonatal transfer compared to babies of adult women.

### 4.1. Prevalence of Maternal Medical and Behavior Characteristics and Birth Outcomes

In this population-based study, 4.7% of births occurred to women younger than 20 years old. Lewis et al. [16] demonstrated that 11% of births in Western Australia were to young women aged 12–18 years. Chen et al. [17] also showed a higher prevalence of births (8.75%) in women <20 years in the United States. The increase of the quality of individual contraceptive counseling and contraceptive knowledge in Russian youth [40] could partly explain our findings on birth prevalence among adolescents. After 1995, the Government of the Russia reduced the benefits to young families, which could also contribute to a postponed parenthood. The postponement of the first birth was attributed to the absence of supportive family policies and economic uncertainty [41].

In our study, the use of 25–29 as a control group was not possible because of the low number of APO that caused a failure of the models to converge. Lewis et al. [16] also treated adults as a whole group to compare pregnancy outcomes between adolescents and adult women. Shrim et al. (2011) [27] compared APO between adolescents and 20–39-years-old women.

Our finding that adolescents were more likely to initiate antenatal care late compared to adult women is supported by the literature [22]. However, direct comparisons of studies are complicated, as the definition of appropriate antenatal care varies. Some studies, like the current one, register GA at the first antenatal visit [25,42] or the number of visits during pregnancy [22,43], while others use more complicated characteristics of antenatal care adequacy that include both the timing and the number of antenatal visits [17,18,20,21] and the infant’s birthweight [15]. The combination of several approaches in one study [22,42] as well as unspecified variables such as “regular visits” [44] have also been reported. Minjares-Granillo et al. (2016) [42] demonstrated a two-week difference in the timing of the first antenatal visit between adolescents and other women, but the results were not statistically significant. Irrespectively of the definition of antenatal care adequacy, the researches mostly demonstrate less adequate care in adolescents compared with adult women [15,17,18,20,21,22,43,44]. In contrast, de Vienne et al. (2009) [45] reported no difference between younger and older women in the studied age groups among 14–30-year-olds.

In our study, the adolescents were more likely to be underweight as well as less likely to be overweight. Earlier studies have consistently shown a higher prevalence of underweight adolescents compared with young adults [15,19]. The prevalence of overweight among adolescents was half of that observed in adults. Torvie et al. (2009) [20] also reported that adolescents had a lower prevalence of overweight compared with women aged 20–24 years.

In our study, twice as many adolescents were smokers compared with adults. We treated adolescents as a whole group, and our findings are consistent with those of the previously published studies of Salihu et al. [15] and Lewis et al. [16]. Chen et al. [17] and Torvie et al. [20], who categorized adolescents in several age groups, specified that the youngest mothers (10–15 and 11–14 years in Chen et al. [17] and Torvie et al. [20], respectively) had a lower prevalence of smoking compared to “older adolescents”. In our study, 3439 (8%) births had no data on smoking, but the proportions of adolescents among those with missing and recorded information on smoking was only 3.5% and 4.8%, respectively. 

We did not find any difference in the prevalence of alcohol abuse between adolescents and adult women. This is consistent with other researchers’ results [15,17,19,42]. Salas-Wright et al. (2015) [46] reported that, compared to their non-pregnant counterparts, pregnant adolescents in the United States were 1.4 and 1.7 times more likely to use alcohol and to have alcohol use disorders, respectively. 

In our study, there were no adolescents with recorded drug abuse. Kawakita et al. (2016) [19] specified that older adolescents (aged 15–19 years) are 1.5 times more likely to be addicted to drugs compared to adults. Salas-Wright et al. (2015) [46] showed that the rate of use of any substances in pregnant adolescents remained 60% higher than in non-pregnant adolescents after adjusting for other studied characteristics, and that young adolescents (12–14 years old) had even a higher rate of substance use compared to older adolescents aged 15–17 years. We did not compare pregnant and non-pregnant adolescents in this study. The ACBR contains information only about pregnant women. Moreover, the information is based on medical records which in turn contains information collected in part as self-reports. 

We found a significantly lower prevalence of folic acid and multivitamin intake in pregnant adolescents compared with adults. These results are consistent with those reported by Branum et al. (2013) [24] who demonstrated a 2.5 times lower prevalence of supplement use among pregnant women aged <25 years compared with older women. Kirbas et al. (2016) [22] reported a lower prevalence of both preconception and prenatal folic acid supplementation in adolescents compared with healthy pregnant women aged 20–34 years. However, two previous studies did not reveal a significant difference in folic acid intake between pregnant adolescents and adults [42,47]. The proportion of missing data was relatively small: 135 (0.3%) and 136 (0.3%) births with missing data on multivitamin and folic acid supplementation, respectively. Compared to adults, the proportion of adolescents was higher among those with missing data for both folic acid (9.4%) and multivitamin (9.6%) intake. Because of the small total number of missing data in these two variables, the final results were unlikely to be affected.

Our finding of a high prevalence of kidney infections in pregnant adolescents (37.5% vs. 33.9% in adults) is consistent with other studies. Vettore et al. (2013) [23] reported that among pregnant women with urinary tract infections the proportion of women aged <20 years (28.4%) was 40% higher than that of women without this health problem. Lewis et al. (2009) [16] also demonstrated that mothers aged 17–18 years experienced a 2.6 times greater risk of urinary tract infection compared to adults. Salihu et al. (2011) [15] reported that the prevalence of renal diseases during the second pregnancy in women aged 10–19 years was higher compared to 20–24-year-olds. The researchers did not specify the variable they used, so we could only suspect that they supposed kidney infections too. 

We found that the prevalence of genital tract infections in adolescents was 50% higher than in adult women. Carter et al. (2011) [48] demonstrated a twice as high proportion of mothers aged <20 years among those who had genital tract infections compared with healthy women. Similarly, the proportions of adolescents (<20 years) with and without genital tract infections among women who gave birth to infants with birth defects were 19.2% and 9.5%, respectively. Chokephaibulkit et al. (1997) [49] demonstrated that 11.2% of pregnant women aged <19 years were infected with *Chlamydia trachomatis*. In contrast to our study, the authors did not investigate older age women. At the same time, we had no information on specific infections to compare our data with those cited above. In our study, the data on both the genital tract and kidney infections were based on medical records that may have a lower risk of misclassification and underreporting compared with self-reports. Since the prevalence of kidney and genital tract infections in our study is relatively high, we are not able to exclude an overdiagnosis in the ACBR records. Carter et al. [48] indicated that, in their study, some mothers might have misreported genital tract infections as urinary tract infections and, in addition, declared only symptomatic diseases, which could lead to a lower prevalence of reported infections.

In the prevalence analyses of APO, we found that, compared to adult women, only neonatal transfer to a higher-level hospital and LBW were significantly higher in adolescents’ infants. Our results are consistent with those of previous studies. Chen et al. (2007) [17] and Torvie et al. (2015) [20] demonstrated that the rate of LBW consistently increased with the decreasing mothers’ age and was the highest among babies born to women aged <15 years. Fayed et al. (2017) [44] demonstrated similar results. Malabarey et al. (2012) [21] also reported a 1.5 times significant difference in LBW proportions between those aged younger than 15 years and those aged 15 years and older. Lewis et al. (2009) [16] found a significant difference in LBW prevalence among adolescents and adult women (24% and 19%, respectively) but did not confirm the difference in the NICU admission prevalence between the studied groups. In addition, the abovementioned study did not show a difference in the prevalence of either NICU admission or LBW between 12–16 and 17–18-year-olds. Shrim et al. (2011) [27] reported a twice as high prevalence of NICU admission in infants born to mothers aged <20 years compared with those born to 20–39-year-olds. As we did not specify how many babies transferred from the delivery units to a higher-level hospital were admitted directly to an NICU, we only could speculate that the proportion of those admitted to an NICU might be higher in adolescents’ newborns compared with older mothers’ babies. In Arkhangelsk County, most severe ill newborns are transported to the NICU at the high-level Regional Children Hospital. Babies with more stable health conditions can be transported to the Pediatric Departments of this or other hospitals. Information about the exact Department where the newborns were transported is partly available in the ACBR.

Although some studies have reported a higher prevalence of stillbirth [16], LBW [50] and VLBW [17], preterm birth <37 [17,19,44] and <32 weeks [17] of gestation, as well as low 5 min Apgar score [16,17,28] in adolescents compared to adult women, we did not identify significant differences of these birth outcomes between the studied age groups. Contrary to studies of other authors, we have not found any difference among adolescents and adult women in APO. 

### 4.2. Risk of APO

We have found that the risk of the most studied APO was similar in adolescents and adult women. In this study, adolescents were at a lower risk of LBW, the 5 min Apgar score <7, and need for neonatal transfer compared with adults. The association of adolescence with APO continues to be a subject of controversy. Our findings are in line with the results of other researchers who have demonstrated no association of maternal young age and stillbirth [15,26,28], VLBW [19], preterm birth [45], or preterm birth at GA < 28 weeks [19]. In our study, the risk of LBW was lower in adolescents compared to adults, which is in contrast with earlier studies [19,45]. Previous studies have not found an increased risk of admission to an NICU [45], or a low Apgar score [19,26,28] in infants born to adolescent women compared to adult women’s babies. In our study, we have found a decreased risk of the 5 min Apgar score <7 and of the need for neonatal transfer in adolescents compared to adults. We have not found an increased risk of perinatal infections in infants, consistent with the results of Kawakita et al. (2016) [19] who reported that the young maternal age treated in age groups was not associated with neonatal pneumonia, sepsis, and meningitis. 

We suggest that early and late adolescence might entail different risks for APO. The stratification by maternal age and the assessment of risk for APO in age groups demonstrate reliable relationships between maternal age and perinatal outcomes. As seen in other studies, the youngest mothers had a higher risk of LBW [25,43,51], preterm birth [19,25,26], stillbirth [45], and low Apgar score [43] compared to older adolescents. Socioeconomic disadvantages for the adolescents are more likely in early age. In this study, we treated adolescents without age grouping, which should be done in the future studies. 

In contrast with the findings of Fraser et al. (1995) [18], some studies [42,51,52] have demonstrated that the socioeconomic status has an impact on the relationship between maternal age and APO. We did not include any economic variables in the multivariable regression models studying the risk of adolescent pregnancy outcomes, because of the limitations of the ACBR database. However, we demonstrated a higher prevalence of smoking, underweight, and late onset of antenatal care in adolescents compared to adults (Table 1). We have found that the inclusion of smoking, underweight, late onset of antenatal care, education, and other potential confounders in all logistic models attenuated the odds of all studied APO, except for perinatal infections in the infants (Table 3). The adjusted odds became significant for LBW, the 5 min Apgar score <7, and the need for neonatal transfer, suggesting confounding effects of the sociodemographic factors.

### 4.3. Strength and Limitations

The strength of this study is a large and validated database. Since the ACBR covers 99.6% of all births that took place in Arkhangelsk County in 2012–2014 [36], the results of our study can be generalized to the entire population of adolescents in Northwest Russia. In addition, the regions in Northwest Russia are similar in their population structure.

In the ACBR, data on smoking, alcohol, and drug abuse during pregnancy were collected from medical records. This attenuates the risk of an under-report bias, but is not able to completely exclude an information bias, as the medical records contain information originally based in part on self-reports. In our study, the proportion of missing information in all studied variables was the smallest in adolescent mothers (data not shown), which could be considered as an advantage. It is likely that the exclusion of the births with missing data on all studied variables did not affect the present study results because of the large sample size.

The finding of a high prevalence of smoking as well as of kidney and genital tract infections in pregnant adolescents compared to the adults could be considered as one additional advantage for health care providers’ and government efforts aimed at the discovery of the risk population.

We did not include data on maternal reproductive history, pre-pregnancy diseases, and complications of pregnancy as potential confounders in the logistic regression analyses, which may be considered as a limitation of this study. However, we adjusted our models for those confounders referring to the association of young maternal age and APO.

## 5. Conclusions

In this study, adolescents gave birth to 4.7% of all infants recorded in the ACBR in 2012–2014. Compared to adults, women aged ≤19 years were more likely to be underweight, to smoke, to miss folic acid and multivitamin intake, and to have kidney and genital tract infections. We did not identify an increased risk of the studied APO in adolescents compared to adults and suppose that factors apart from young maternal age contribute more to APO. We found a lower risk of LBW, the 5 min Apgar score <7, and the need for neonatal transfer in adolescents’ infants. The results of this study may be useful in generating and implementing prevention and intervention strategies in Northwest Russia. Our findings may be also a benefit for doctors and midwives planning their work when dealing with pregnant adolescents. Particular efforts should be undertaken in order to increase the multivitamin and folic acid supplementation rate in pregnant adolescents as well as to provide more effective measures to prevent genital tract infections and promote a healthy life style in adolescents. 

## Figures and Tables

**Table 1:** Bivariate analyses of maternal medical and behavior characteristics, according to mothers’ age, the ACBR, Russia, 2012–2014.

Maternal Characteristics	*n* Without Missing Information	Adolescents *n* (%)	Adult Women *n* (%)	*p*-Value *
Late antenatal visit	42,855	614 (30.7)	5148 (12.6)	<0.001
BMI, kg/m^2^	42,751			<0.001
<18.5		219 (11.0)	2604 (6.4)	
18.5–24.9		1465 (73.3)	25,985 (63.8)	
≥25		314 (15.7)	12,164 (29.8)	
Smoking	39,888	604 (31.6)	5875 (15.5)	<0.001
Evidence of alcohol abuse	43,318	7 (0.3)	171 (0.4)	0.631
Evidence of drug abuse	43,320	0 (0.0)	14 (0.0)	0.406
Folic acid intake	43,191	866 (42.9)	22,117 (53.7)	<0.001
Multivitamin intake	43,192	961 (47.6)	22,332 (54.2)	<0.001
Infections of kidney in pregnancy ^1^	43,327	762 (37.5)	13,980 (33.9)	0.001
Infections of the genital tract in pregnancy ^2^	43,327	495 (24.3)	6600 (16.0)	<0.001
Parity (para)	39,249	160 (8.9)	21,553 (57.6)	<0.001

ACBR: Arkhangelsk County Birth Registry; *n*: number; BMI: body mass index. * *p* for chi-squared tests. ^1^ ICD-10 code O23.0; ^2^ ICD-10 code O23.5.

**Table 2:** Bivariate analyses of adverse pregnancy outcomes, according to mothers’ age, the ACBR, Russia, 2012–2014.

Perinatal Outcomes	*n* Without Missing Information	Adolescents *n* (%)	Adult Women *n* (%)	*p*-Value *
Stillbirth	42,757	18 (0.9%)	279 (0.7%)	0.278
Preterm birth (<37 weeks)	42,633	23 (1.1%)	530 (1.3%)	0.540
Preterm birth (<32 weeks)	42,633	140 (7.0%)	2452 (6.0%)	0.085
Neonatal transfer to a higher-level hospital	42,831	227 (11.2%)	4012 (9.8%)	0.042
Low birthweight	42,824	133 (6.6%)	2158 (5.8%)	0.012
Very low birthweight	42,824	18 (0.9%)	413 (1.0%)	0.589
Perinatal infections in infants	42,833	5 (0.2%)	43 (0.1%)	0.063
The 5 min Apgar score <7	42,425	36 (1.8%)	778 (1.9%)	0.694
The 5 min Apgar score <4	42,425	14 (0.7%)	232 (0.6%)	0.467

ACBR: Arkhangelsk County Birth Registry; *n*: number. * *p* for chi-squared tests.

**Table 3:** The crude and adjusted odds ratios for adverse pregnancy outcomes in adolescents, the ACBR, Russia, 2012–2014.

Perinatal Outcomes	*n* (*n*_1_)	Unadjusted ORs	95% CI	Adjusted * ORs	95% CI
Stillbirth	35,066 (232)	1.30	0.81, 2.10	0.88	0.49, 1.58
Preterm birth (<37 weeks)	35,110 (2095)	1.17	0.98, 1.39	0.94	0.76, 1.17
Preterm birth (<32 weeks)	35,110 (438)	0.88	0.58, 1.34	0.66	0.39, 1.11
Neonatal transfer to a higher-level hospital	35,110 (3412)	1.16	1.01, 1.34	0.81	0.68, 0.96
Low birthweight	35,104 (1872)	1.26	1.05, 1.51	0.72 **	0.55, 0.95
Very low birthweight	35,104 (348)	0.88	0.55, 1.41	0.58 **	0.30, 1.09
Perinatal infections in infants	35,224 (40)	2.35	0.93, 5.93	3.05	0.92, 10.12
The 5 min Apgar score <7	34,799 (640)	0.94	0.67, 1.31	0.55	0.37, 0.84
The 5 min Apgar score <4	34,799 (184)	1.22	0.71, 2.10	0.64	0.33, 1.27

ACBR: Arkhangelsk County Birth Registry; *n*: number of births included in the final regression model; *n*_1_: number of studied outcomes in the final regression model; ORs: odds ratio; CI: confidence intervals. * Adjusted for maternal body mass index, education, smoking, year of delivery, multivitamin and folic acid intake, infections of the kidney and the genital tract in pregnancy, timing of the first antenatal visit, parity; ** Adjusted for all variables and potential confounders listed above and preterm birth (<37 weeks of gestation).
